# Bacterial Polyglucuronic Acid/Alginate/Carbon Nanofibers Hydrogel Nanocomposite as a Potential Scaffold for Bone Tissue Engineering

**DOI:** 10.3390/ma15072494

**Published:** 2022-03-28

**Authors:** Zahra Ebrahimvand Dibazar, Mahnaz Mohammadpour, Hadi Samadian, Soheila Zare, Mehdi Azizi, Masoud Hamidi, Redouan Elboutachfaiti, Emmanuel Petit, Cédric Delattre

**Affiliations:** 1 Department of Oral and Maxillo Facial Medicine, Faculty of Dentistry, Tabriz Asad University of Medical Sciences, Tabriz 5166616471, Iran; dibazarz@yahoo.com; 2 Department of Chemistry, Faculty of Sciences, Tarbiat Modares University, Tehran 1411713116, Iran; mahnazmo-hammadpour@modares.ac.ir; 3 Pharmaceutical Sciences Research Center, Health Institute, Kermanshah University of Medical Sciences, Kermanshah 6715847141, Iran; 4 Student Research Committee, Zanjan University of Medical Sciences, Zanjan 7797845157, Iran; zare.soheila.1150@zums.ac.ir; 5 Department of Tissue Engineering and Biomaterials, School of Advanced Medical Sciences and Technologies, Hamadan University of Medical Sciences, Hamadan 6517838636, Iran; m.azizi@umsha.ac.ir; 6 BioMatter-Biomass Transformation Lab (BTL), École Polytechnique de Bruxelles, Université Libre de Bruxelles, Avenue F.D. Roosevelt, 50-CP 165/61, 1050 Brussels, Belgium; masoud.hamidi@ulb.ac.be; 7 Department of Medical Biotechnology, Faculty of Paramedicine, Guilan University of Medical Sciences, Rasht 4188794755, Iran; 8 UMRT INRAE 1158 BioEcoAgro, Laboratoire BIOPI, Université de Picardie Jules Verne, IUT d’Amiens, 80025 Amiens, France; emmanuel.petit@u-picardie.fr; 9 Université Clermont Auvergne, Clermont Auvergne INP, CNRS, Institut Pascal, 63000 Clermont-Ferrand, France; 10 Institut Universitaire de France (IUF), 1 Rue Descartes, 75005 Paris, France

**Keywords:** polyglucuronic acid, *Sinorhizobium meliloti* M5N1CS, carbon nanofibers, hydrogel, bone tissue engineering, nanocomposites

## Abstract

3D nanocomposite scaffolds have attracted significant attention in bone tissue engineering applications. In the current study, we fabricated a 3D nanocomposite scaffold based on a bacterial polyglucuronic acid (PGU) and sodium alginate (Alg) composite with carbon nanofibers (CNFs) as the bone tissue engineering scaffold. The CNFs were obtained from electrospun polyacrylonitrile nanofibers through heat treatment. The fabricated CNFs were incorporated into a PGU/Alg polymeric solution, which was physically cross-linked using CaCl_2_ solution. The fabricated nanocomposites were characterized to evaluate the internal structure, porosity, swelling kinetics, hemocompatibility, and cytocompatibility. The characterizations indicated that the nanocomposites have a porous structure with interconnected pores architecture, proper water absorption, and retention characteristics. The in vitro studies revealed that the nanocomposites were hemocompatible with negligible hemolysis induction. The cell viability assessment showed that the nanocomposites were biocompatible and supported bone cell growth. These results indicated that the fabricated bacterial PGU/Alg/CNFs hydrogel nanocomposite exhibited appropriate properties and can be considered a new biomaterial for bone tissue engineering scaffolds.

## 1. Introduction

Bone regeneration is a complex and multifactorial process that may require intervention in large defects [1]. The allografts are the gold standard treatment options, providing acceptable treatment results. Despite the acceptable finding, this method suffers from some critical limitations, such as a low source of allografts, painful donor site harvesting surgery, and donor site morbidity. Accordingly, novel and innovative approaches must be proposed and developed as the alternative over the allograft implantation. Tissue engineering is a multi/interdisciplinary approach integrating materials sciences, biology, and engineering concepts to develop techniques enhancing tissue regeneration [2,3,4].

Scaffolds as the central part of bone tissue engineering have attracted significant attention, and a large number of studies have been conducted to design, fabricate, modify, and optimize scaffolds with the highest similarity to native tissues. 3D scaffolds have shown promising results in bone regeneration [5,6]. Hydrogels are 3D structures fabricated from hydrophilic polymers, cross-linked using proper methods, and can absorb and maintain a large amount of water without dissolving in water. The swollen nature and cross-linked polymer chains of hydrogels provide a 3D and porous architecture, which is beneficial for bone tissue engineering applications. The porous structure is critical for nutrient/waste turnover, as well as cell migration and infiltration. Various types of synthetic and natural polymers can be applied for hydrogel fabrication. Natural polymers provide biodegradation and lower toxicity issues than synthetic polymers do [7,8,9].

Polysaccharides-based natural polymers have functional and structural diversity and have promising potential as tissue engineering scaffolds [10,11,12,13]. In tissue engineering, alginate (Alg) is the most commonly utilized natural polysaccharide for hydrogelation [14,15,16]. Alg, like chitin and chitosan, is frequently employed for bone tissue engineering [16]; for example Alg hydrogels are employed as fillers and carriers of osteoinductive factors [17,18]. This is due to the fact that Alg has several desirable characteristics, including water solubility, biocompatibility, low toxicity, low cost, extended active agent release and moderate and quick hydrogelation when divalent cations, such as Ca^2+^, are added [18,19,20,21]. However, natural polymers such as Alg have limits due to mechanical qualities that are insufficient to be used in bone tissue engineering [17,18,20,21]. To address these issues, a variety of materials, such as nanomaterials, have been mixed into the alginate structure, resulting in strong composite materials [22,23,24].

Nanotechnology and nanomaterials provide the possibility to design, fabricate, and modify structures with the highest precision and orientation to the intended applications. Nanostructured bone tissue engineering scaffolds have shown fascinating results in bone regeneration due to their high similarity to the bones’ native structure [25,26,27,28]. Nanofibers possess a high surface-to-volume ratio and resemblance to the extracellular matrix (ECM) of bone and, accordingly, have the highest potential to be applied as the scaffold or a component of bone tissue engineering scaffolds. Different methods have been developed for nanofibers fabrication. Among them, electrospinning has attracted unprecedented attention due to its flexibility, low fabrication cost, and versatility for fabricated nanofibers with different morphology and architecture from a wide range of natural, synthetic, and semisynthetic polymers [29,30,31,32]. The conductive fibers with or without electrical stimuli have attracted widespread attention in recent years due to their ability to mediate a variety of cellular responses. On the other hand, electrical stimulation has long been recognized for its role in cell motility and differentiation. As a result, including electroconductive components such as carbon nanofibers (CNFs) in scaffolds promotes the development and signaling of neighboring cells [33]. For instance, Serafin et al. hybrid developed an electroconductive and printable 3D scaffolds consisting of alginate and gelatin hydrogel filled with CNFs [34]. In this present study, carbon nanofibers (CNFs) were used because they have additional advantages over the other polymeric nanofibers, such as high mechanical strength and acceptable electrical conductivity [35]. Moreover, our previous studies showed that the CNF scan supports bone cell attachment and proliferation [36]. Electrospun CNFs are fabricated from precursor polymeric nanofibers (e.g., polyacrylonitrile (PAN)) under heat treatment. Implementation of CNFs into the 3D hydrogel as the filler to fabricate the hydrogel nanocomposite integrates the beneficial properties of hydrogel with the nano-feature, electrical, and mechanical properties of CNFs [37,38].

The search for new biomaterials for tissue engineering, microbial exopolysaccharides (EPSs) have received great attention in recent years. Microbial EPSs such as bacterial Alg are produced by a variety of bacteria and fungi and are involved in a variety of physiological processes, including biofilm construction, nutrition acquisition, stress resistance, and antimicrobial resistance [39,40]. In this particular context, a polyglucuronic acid called glucuronan (PGU), as an EPS produced by the *Sinorhizobium meliloti* M5N1CS strain, a Gram-negative α-proteobacterium capable of fixing atmospheric nitrogen, described a high molecular weight anionic homopolysaccharide made up of →(4)-*β*-D-GlcpA-(1)→residue partially *O*-acetylated at the C-3 and/or the C-2 position [10,39,41,42,43].

This bacterial EPS is a biocompatible, non-cytotoxic, and bioactive biopolymer used in cosmetics and pharmaceuticals as a hydrating, thickening, and gelling agent. PGU was recently considered for tissue engineering applications as a very promising polysaccharides-based biomaterial used in functional tissue fabrication [10,44] and could be efficiently used and investigated for new applications in tissue engineering, as already done with alginate [44], and can be utilized as an alternative for pectin and alginate in the cosmetic and pharmaceutical industries [42,45,46,47]. Furthermore, the immunostimulating properties of PGU and oligo-PGU were mentionned in a French patent (FR2781673), and they have been demonstrated on human blood monocytes to be better productions of Il-6 and TNF-α cytokines and equivalent productions of Il-1 cytokine in comparison with alginate. Therefore, PGU can have an important role as a reconstructive biomaterial that could help in healing the residual cavity [48], stop bleeding during surgery, prevent the formation and reformation of post-surgical adhesions, and promote bone regeneration [49].

Therefore, the main objective of the present assay was to fabricate 3D hydrogel nanocomposites based on PGU/Alg hydrogel integrated with CNFs as the bone tissue engineering scaffold.

## 2. Materials and Methods

### 2.1. Materials

Polyacrylonitrile (PAN) (Mw = 150,000 g/mol) was acquired from Polyacryl Iran Corporation (Isfahan, Iran). The crosslinker used the chloride salt of calcium (CaCl_2_·4H_2_O), and sodium alginate (alginic acid sodium salt), which were obtained from Merck (Darmstadt, Germany). Trypsin-EDTA, Penicillin–Streptomycin (Pen–Strep), a DMEM/F-12 cell culture medium, and fetal bovine serum (FBS) were purchased from Gibco (Darmstadt, Germany). The MG-63 cell line was from the Pasteur Institute, Tehran, Iran. Plastics and tissue culture plates were from SPL, Gyeonggi-do, Korea. All commercial starting materials and solvents in this work were analytical grade and were used as supplied and without any further purification, unless otherwise specified. 

### 2.2. Polyglucuronic Acid (PGU) Production

Partially acetylated anionic bacterial glucuronan (PGU) production was carried out in a 20 L bioreactor (SGI, La Chapelle-Saint-Luc, France) for 72 h at 30 °C and 200 rpm. The bioreactor was filled with 15 L of rhizobium complete medium supplemented with 1% (*v*/*v*) sucrose as a carbon source (RCS). The inoculation was performed with 10% (*v*/*v*) of *Sinorhizobium meliloti* M5N1CS culture in the exponential phase of growth. The pH was regulated to 7.2 with 4 mol/L KOH solution. After 72 h, the bacteria were removed from the broth by centrifugation at 33,900 × *g* for 40 min at 20 °C. The clarified supernatant was purified, then concentrated by tangential flow ultrafiltration on a 100,000 normal-molecular-weight cutoff (NMWCO) polyethersulfone membrane from Sartorius (Goettingen, Germany) against distilled water. The finally purified supernatant was freeze-dried to give a white dry layer of glucuronan.

### 2.3. Preparation of Carbon Nanofiber (CNF)

According to the procedure reported previously [36,50], 500 mg of PAN powder were dissolved in 5 mL of DMF to prepare a 10% *w*/*v* solution. The medium was heated to 45 °C and stirred for 12 h until a homogeneous solution was obtained. A commercial electrospinning setup (Fanavaran Nano Meghyas Ltd., Co., Tehran, Iran) comprised of a syringe pump, a rotating collector drum, and a high voltage power source was employed to fabricate nanofibers. The PAN solution was placed in a 5 mL syringe with an 18-gauge metal tip as the nozzle and then was electrospun with a 0.5 mL/h feed rate and 20 kV of fixed DC voltage onto the aluminum foil-covered collector at a distance of 15 cm from the needle tip. Eventually, the formed nanomats were carefully removed from the aluminum foil. 

Electrospun PAN nanofibers, as primary precursors of CNF, were stabilized via heat treatment of the nanofibers at 290 °C for 4 h in an air atmosphere (with the heating ramp of 1.5 °C/min). Subsequently, the carbonization process was performed in a tube furnace by heating the stabilized nanofibers at 1000 °C with a heating ramp of 4 ºC/min to reach 1000 °C and further heating at 1000 °C for 1 h.

### 2.4. CNFs Characterization 

A scanning electron microscope (SEM; FEI Model Quanta 450, Hillsboro, OR, USA) was applied to image the morphology of the fabricated nanofibers (PAN and the final CNFs). The nanofibers were coated with a thin layer of conducting element (gold) using a sputter-coater and imaged at the accelerating voltage of 20 kV. The diameter of the nanofibers was measured/calculated using ImageJ software (Version: 1.4.3.6.7, NIH). The microstructure of the CNFs was assessed using Raman spectroscopy. The experiment was conducted using a Teksan Takram P50C0R10 Raman spectrometer (Tehran, Iran) with the laser power of 0.5–70 mW and a laser wavelength of 532 nm. The crystallinity of CNFs was evaluated using the X-ray Diffraction (XRD) technique. The XRD pattern was recorded by a Siemens D5000 diffractometer (Aubrey, TX, USA) (Cu Kα radiation with λ = 1.5406 Å, 40 kV, 30 mA, 2θ range from 10° to 80° and the step size of 0.05°/s).

### 2.5. Production of Alg/PGU/CNFs Hydrogels

Amounts of 15, 30, and 45 mg of CNF were suspended in 10 mL of deionized water. The obtained suspensions were magnetically stirred for 1 h and re-dispersed by an ultrasonic bath several times at the same time. Then 100 mg (1% *w*/*v*) of PGU were added into each mixture and stirred at 40 °C until the extracts were completely dissolved. After that, 200 mg of sodium alginate were added to the reaction vessels and further stirred at 40 °C for another 3 h to ensure system uniformity. Then, CaCl_2_ (100 mmol) was added to the solution (1:1) and incubated for 24 h to fabricate the hydrogel nanocomposite. 

### 2.6. Hydrogel Nanocomposite Characterization 

SEM was used to visualize the internal structure of the scaffold and pores architecture. The scaffolds were frozen at −20 °C for 24 h and freeze-dried using a freeze dryer for 48 h. Then, the scaffolds were crashed and coated with a thin layer of gold and imaged using an SEM (SEM; FEI Model Quanta 450, Hillsboro, USA) device. The pore size of the scaffolds was measured using ImageJ software (NIH). 

The liquid displacement method was applied to measure the porosity of the prepared scaffolds. Briefly, the initial weight of absolute ethanol was measured (W1); a specific among the hydrogel nanocomposites was submerged for one h; the weight change was recorded (W2); and, finally, the scaffold was removed and the ethanol weight change was recorded again (W3). Equation (1) was used to calculate the porosity.

(1) $$Porosity\;\left(\%\right)\;=\;\left(W1-W3/W2-W3\right)\times 100$$

The profile of water absorption capacity versus time of the fabricated hydrogel nanocomposites was obtained using the gravimetric method. The scaffolds were weighed and then soaked in PBS (pH 7.4) at ambient conditions for 96 h. The samples were removed from the solution at the predetermined time points and weighed again. Equation (2) was used to calculate the water absorption capacity.

(2) $$Water\;absorption\;capacity\;\left(\%\right)\;=\;\left(\frac{W1-W0}{W0}\right)\times 100$$

The weight loss of the fabricated scaffold in PBS solution was measured as the indication of biodegradability. The scaffolds were weighed and incubated in PBS solution for two weeks at 37 °C. At the specific time points, the samples were removed, dried, and weighed again to calculate the weight loss percentage. 

### 2.7. In Vitro Assessments 

#### 2.7.1. Hemolysis Induction Assay 

The fabricated scaffolds (50 mg) were incubated with 200 μL of fresh and anticoagulated blood (diluted with PBS, 2:2.5) at 37 °C for 1 h. Then, the samples were centrifuged at 1500 rpm for 3 min at 40 °C. Finally, the absorbance of the supernatant was read at 545 nm using a microplate reader. Equation (3) was applied to calculate the hemolysis percentage:

(3) $$Hemolysis\;\left(\%\right)\;=\;\left(\frac{Dt-Dnc}{Dpc-Dnc}\right)\times 100$$

where *Dt* = absorbance of the sample, *Dnc* = absorbance of the negative control, and *Dpc* = absorbance of the positive control.

#### 2.7.2. Cell Viability 

The MTT assay kit was used to measure the viability of MG-63 cells on/into the fabricated hydrogel nanocomposites. Scaffolds were placed at the bottom of a 96-well tissue culture plate and sterilized using ethanol (70%) following several washes with autoclaved PBS and UV exposure for 2 h. Then, the cells (5000/well) were suspended in a cell culture medium containing FBS (10% *v*/*v*) and antibiotics (Pen/Strep) and incubated with the scaffolds at 37 °C with 5% CO_2_ in a cell culture incubator for 24 and 72 h. At the end of the time points, 150 μL of MTT solution (0.5 mg/mL) were added to each well and incubated for another 4 h at 37 °C in a dark place. Finally, the formazan crystals were dissolved using 100 μL of DMSO, and the absorbance of the solutions was read using a microplate reader at 540 nm, and the cell viability was calculated using Equation (4). The control group was cells in wells without a scaffold.

(4) $$Cell\;viablity\;\left(\%\right)\;=\;\left(\frac{Dt-Dc}{Dc}\right)\times 100$$

*Dt* is the absorbance of the test, and *Dc* is absorbance of the control. 

### 2.8. Statistical Analysis

The SPSS software (version 10.0) was used to calculate the statistical analysis of the obtained results. The experiments were conducted in triplicate, and the results were reported as a mean ± SD with a significance level of *p* < 0.05.

## 3. Results

### 3.1. PAN and CNFs Characteristics

**Figure 1:**
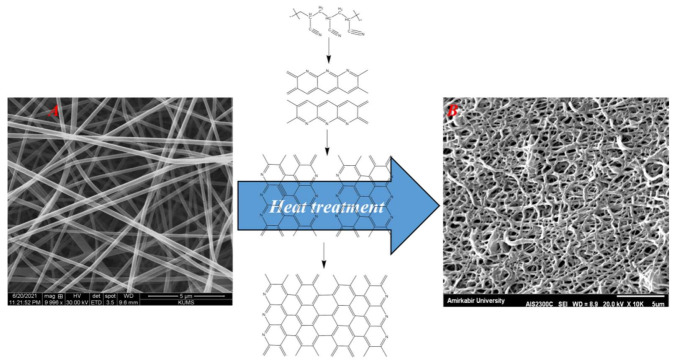
SEM images of PAN (**A**) and CNFs (**B**).

The morphology of the fabricated PAN and CNFs was visualized using an SEM imaging technique, and the results are presented in Figure 1. The results showed that the PAN nanofibers were straight and beadles (Figure 1A), while the CNFs were fused with transverse connections (Figure 1B). Measuring the diameter of PAN nanofibers showed that the nanofibers have a diameter of 233 ± 30 nm, and CNFs have a diameter of 202 ± 60 nm. 

The crystallinity and microstructure of the synthesized CNFs were evaluated using XRD and Raman analysis, and the obtained quantitative data are presented in Table 1. The results revealed that the synthesized CNFs possess graphene nanocrystals in an amorphous graphitic structure.

### 3.2. Hydrogel Nanocomposite Characteristics

#### 3.2.1. Internal Structure and Morphology 

**Figure 2:**
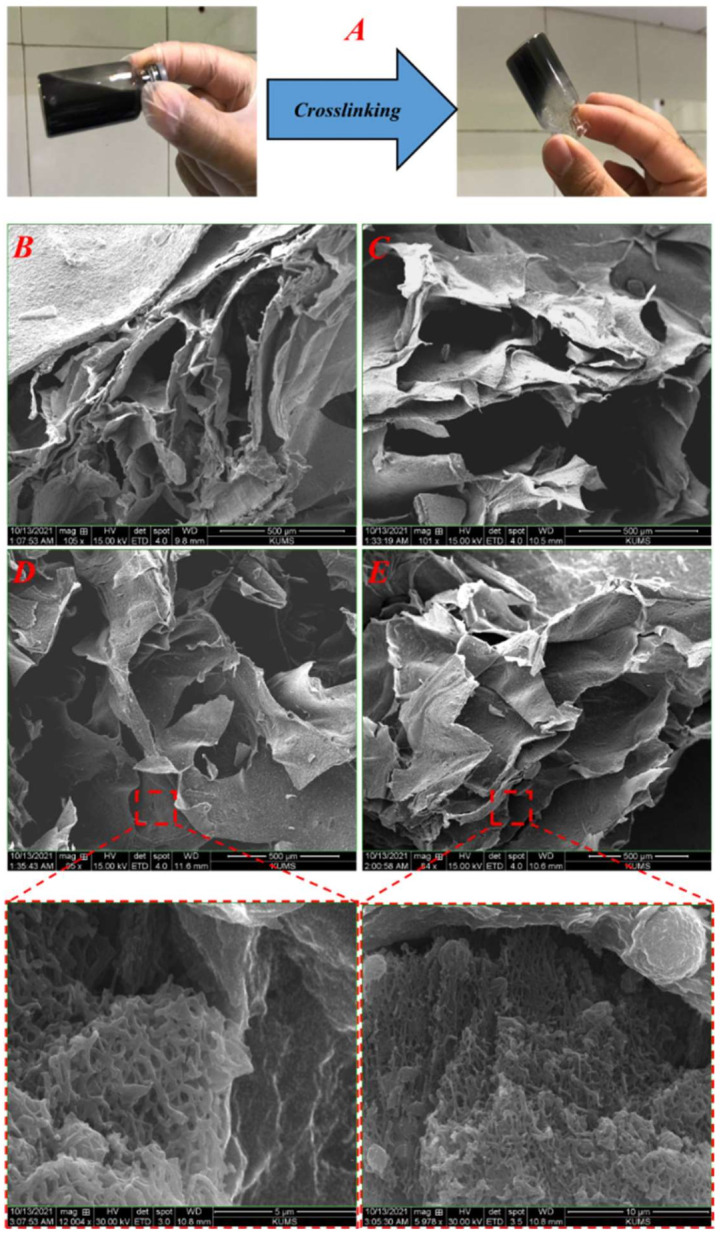
(**A**) Macroscopic image of the hydrogel formation upon the cross-linking, (**B**) SEM image of PGU/Alg hydrogel, (**C**) SEM image of PGU/Alg/CNFs 5%, (**D**) SEM image of PGU/ALg/CNFs 10%, and (**E**) SEM image of PGU/Alg/CNFs 15% (**D**).

The internal structure and pores architecture were visualized using the SEM imaging method, and the results are presented in Figure 2. As can be seen, the polymeric solution is cross-linked to a gel under the cross-linking process (Figure 2A). Moreover, the fabricated hydrogel nanocomposites possess a porous microstructure with interconnected pores (Figure 2B–E). Furthermore, the presence of CNFs in the polymeric matrix of the hydrogel can be seen (inserts in Figure 2D,E).

#### 3.2.2. Swelling Kinetics 

**Figure 3:**
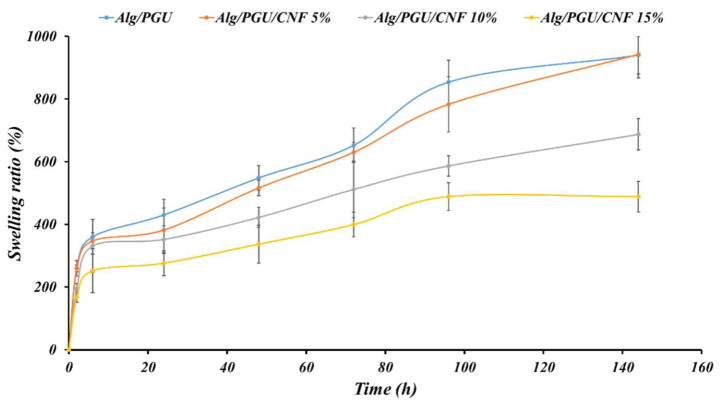
Swelling of the prepared hydrogel nanocomposites measured based on the gravimetric method.

Hydrogels’ swelling capacity is an important parameter contributing to their application in bone regeneration. In addition, scaffolds hydrogel used in vivo will be rapidly exposed to tissue fluids and absorb water into their polymeric matrix structures. The water absorbed promotes the expansion of porous architecture, which in turn facilitates cell entry, the diffusion of nutrients, and the excretion of metabolites. In this work, we studied the hydration/swelling ability of our hydrogels by immersing them in the PBS (pH 7.4) at ambient conditions. The results in Figure 3 show that PGU/Alg hydrogel composites without CNFs possess the ability to absorb and retain water more than their original weight. The presence of hydroxyl (-OH) and carboxyl (-COOH) groups in PGU/Alg backbones offers many opportunities to create multiple hydrogen bonds with water molecules. However, it is known that uncontrolled swelling can cause adverse effects on the mechanical property of the implanted PGU/Alg hydrogels scaffolds and disruption of pores architecture and can provoke surrounding tissue deformation, inducing pain to the patient. The incorporation of carbon nanofibers CNFs in the PGU/Alg hydrogel matrix can influence the swelling capacity and help to avoid system disintegration. In this study, the introduction of CNFs into PGU/Alg hydrogel scaffolds in the increasing order of concentration significantly reduced the swelling ability of the PGU/Alg/CNFs scaffolds. Figure 3 showed that PGU/Alg/CNFs (15%) generate a reduced swelling ability of over half compared to PGU/Alg hydrogels composites. 

The decrease in the swelling capacity of the PGU/Alg hydrogel in the presence of CNFs is possibly due, firstly, to the fact that CNFs absorbed only a small amount of water through –OH and –NH_2_ groups. Secondarily, carbon nanofibers CNFs in the PGU/Alg hydrogel system acted as a multifunctional cross-linker generating a network system opposing the expansion of the hydrogel composite. 

#### 3.2.3. Degradation Results 

**Figure 4:**
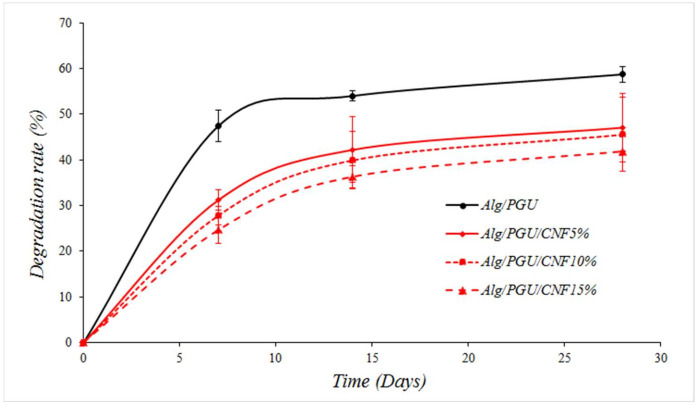
Weight loss values of the prepared scaffolds in PBS solution over 28 days.

The weight loss percentage of the fabricated scaffolds in PBS solution was measured as the indication of biodegradation. The results showed that the incorporation of CNFs reduced the weight loss values. The highest weight loss was obtained in the CNFs-free scaffold, and the lowest weight loss resulted from the scaffold with the highest CNFs concentration (15%) (Figure 4). 

### 3.3. In Vitro Assessments Results 

#### 3.3.1. Hemocompatibility 

**Figure 5:**
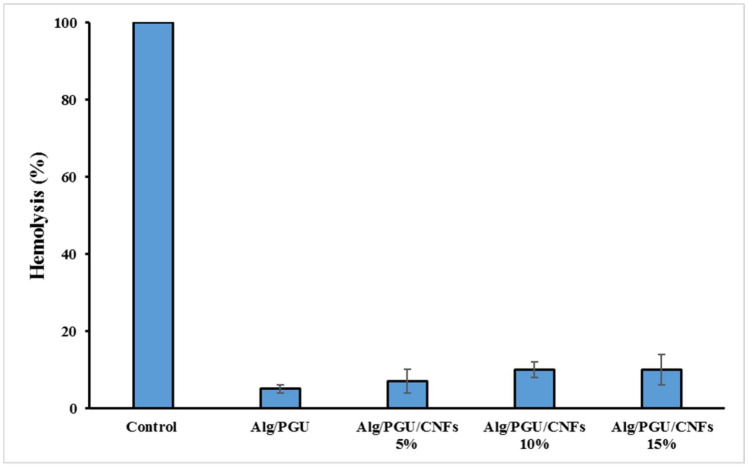
The hemolysis induced by the fabricated scaffolds.

Hemolysis induced by the scaffolds was evaluated as the indication of the hemocompatibility of the scaffolds. The results implied that the fabricated scaffolds did not induce significant hemolysis and can be considered hemocompatible structures (Figure 5). 

#### 3.3.2. Cell Viability 

**Figure 6:**
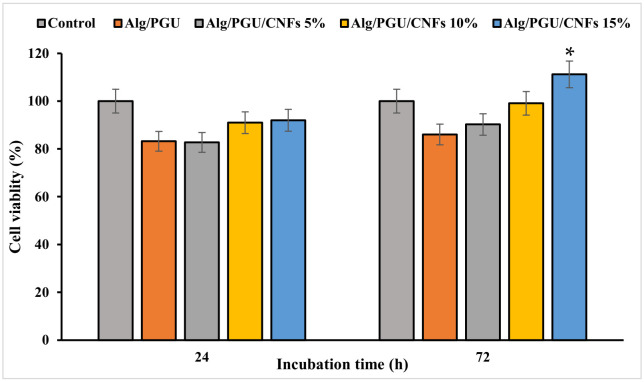
Viability of MG-63 cell on the prepared hydrogel nanocomposites measured by the MTT assay kit. Values represent the mean ± SD, *n* = 5, * *p* < 0.05 (obtained by one-way ANOVA).

The viability of MG-63 cells on the fabricated hydrogels was measured using the MTT assay kit, and the results are presented in Figure 6. The results revealed that the fabricated scaffolds were cytocompatible and did not induce significant toxicity. The hydrogel with the highest concentration of CNFs was not only cytocompatible but also induced a proliferative effect, which was statistically significant compared to the control (*p* < 0.05). 

## 4. Discussion

Scaffolds have inevitable and determinant roles in tissue engineering approaches by providing a substrate for cells attachment, growth, migration, and differentiation. Hydrogels have shown promising results as bone tissue engineering scaffolds due to their 3D structure, high porous profile, high water absorption capacity, and interconnected pores structure. For a sophisticated tissue engineering strategy, it is critical to implement more physical and biological features within hydrogels. Nanomaterials can be implemented into the hydrogel to precisely tailor the internal structure of the scaffold. The incorporation of nanofibers confers ECM similarity to the hydrogel and provides cell attachment sites with high surface areas [34,51,52,53,54,55,56]. 

CNFs possess electrical conductivity and mechanical strength along with morphological advantages. Accordingly, the main objective of the current study is to fabricate hydrogels based on PGU/Alg composites with CNFs as the bone tissue engineering scaffold [34]. The CNFs were obtained from polyacrylonitrile (PAN) nanofibers using heat treatment. The SEM image of nanofibers showed heat treatment-induced nanofibers fusion and reduced nanofibers uniformity. These phenomena can be due to chemical changes during heat treatment. Moreover, the diameter of the CNFs was lower than those of the PAN nanofibers, which is due to the removal of non-carbonaceous substances from PAN nanofibers and the shrinkage of the nanofibers [36]. The fabricated CNFs possess a network/fused structure, and it is impossible to convert them to individual nanofibers. Alternatively, we crashed the CNFs mat to micrometric clusters/aggregates of CNFs rather than individual nanofibers and dispersed the clusters/aggregates into the polymeric solution. Then, the clusters/aggregates were incorporated into the matrix of the hydrogel through physical entrapment during the cross-linking. 

The hydrogel morphology evaluation showed that they have porous structures beneficial for bone tissue engineering. It has been reported that the porous scaffolds provide space for bone cells migration throughout the deepest parts of scaffolds, and their colonization improves the success of bone regeneration. On the other hand, the cells’ growth and colonization are limited to the surface of the non-porous scaffolds, which fails the treatment approach [57,58]. The swelling kinetic evaluation revealed that the incorporation of CNFs reduced the swelling capacity of the hydrogel. This may be due to the larger amount of polymer chains interactions induced by CNFs. The higher interactions prevent the swelling of the hydrogel. However, the observed swelling of all scaffolds with different contents of CNFs is high enough to provide a high content of water and induce porous nature.

The biological evaluations also depicted that the fabricated scaffolds were hemocompatible (negligible hemolysis) and cytocompatible. The highest cell proliferation was obtained using the hydrogel containing 15% CNFs. These findings revealed that fabricated scaffolds can be applied as the bone tissue engineering scaffold.

## 5. Conclusions

Nanomaterials-enabled structures have shown fascinating performance in various fields, such as biomedicine. It is possible to precisely design, fabricate, and modify structures using nanomaterials and nanotechnology. CNFs are engineered nanomaterials with outstanding properties. In the current paper, we have shown that it is possible to synthesize PAN-derived CNFs and integrate them into polysaccharide-based hydrogels as the 3D nanocomposite scaffold. Using this approach, we integrated the biological and 3D structure of a PGU-based hydrogel with the nanofibrous feature of CNFs, and the results revealed that the resulting scaffold exhibited promising properties beneficial for bone tissue engineering applications. However, it is critical to evaluate the expression of bone-related gene profiles under treatment with the scaffolds, in vitro and in vivo. Moreover, conducting long-term in vitro and in vivo studies can help to clarify the fate and toxicity of the fabricated scaffolds. Further investigations that support the capacity of this new and innovative biomaterial will be performed to promote in vivo bone regeneration for greater completeness, including the mechanical properties, intermolecular forces, and biodegradability of the bacterial polyglucuronic acid/alginate/carbon nanofiber hydrogel nanocomposite. 

## Figures and Tables

**Table 1:** Quantitative results of Raman and XRD analysis.

XRD Results							
Lc (nm)		d (002) Å		FWHM		2θ	
4.7		3.26		0.19		26.32	
**Raman Results**							
**D Position (cm^−1^)**	**G Position (cm^−1^)**	**ID/IG (integrated areas)**	**Crystallite Parameters**		**Defect Parameters**		**R(ID/IG)**
			**Size La (nm)**	**Area** **La^2^ (nm^2^)**	**Average Distanse L_D_(nm)**	**Density n_D_(cm^−2^ × 10^11^)**	
1372.14	1582.42	3.41	5.63	31.69	2.05	7.65	0.97

## Data Availability

Further data are available upon reasonable request from the corresponding author.
